# Effect of a low fat versus a low carbohydrate weight loss dietary intervention on biomarkers of long term survival in breast cancer patients ('CHOICE'): study protocol

**DOI:** 10.1186/1471-2407-11-287

**Published:** 2011-07-06

**Authors:** Scot M Sedlacek, Mary C Playdon, Pamela Wolfe, John N McGinley, Mark R Wisthoff, Elizabeth A Daeninck, Weiqin Jiang, Zongjian Zhu, Henry J Thompson

**Affiliations:** 1 Cancer Prevention Laboratory, Colorado State University, Fort Collins, CO, USA; 2 Rocky Mountain Cancer Centers, Denver CO, USA; 3 Colorado Biostatistics Consortium, University of Colorado, Denver, CO, USA

**Keywords:** biomarkers, dietary patterns, low fat, low carbohydrate, weight loss, breast cancer, long term survival

## Abstract

**Background:**

Weight loss in overweight or obese breast cancer patients is associated with an improved prognosis for long term survival. However, it is not clear whether the macronutrient composition of the chosen weight loss dietary plan imparts further prognostic benefit. A study protocol is presented for a dietary intervention to investigate the effects of weight loss dietary patterns that vary markedly in fat and carbohydrate contents on biomarkers of exposure to metabolic processes that may promote tumorigenesis and that are predictive of long term survival. The study will also determine how much weight must be lost for biomarkers to change in a favorable direction.

**Methods/Design:**

Approximately 370 overweight or obese postmenopausal breast cancer survivors (body mass index: 25.0 to 34.9 kg/m^2^) will be accrued and assigned to one of two weight loss intervention programs or a non-intervention control group. The dietary intervention is implemented in a free living population to test the two extremes of popular weight loss dietary patterns: a high carbohydrate, low fat diet versus a low carbohydrate, high fat diet. The effects of these dietary patterns on biomarkers for glucose homeostasis, chronic inflammation, cellular oxidation, and steroid sex hormone metabolism will be measured. Participants will attend 3 screening and dietary education visits, and 7 monthly one-on-one dietary counseling and clinical data measurement visits in addition to 5 group visits in the intervention arms. Participants in the control arm will attend two clinical data measurement visits at baseline and 6 months. The primary outcome is high sensitivity C-reactive protein. Secondary outcomes include interleukin-6, tumor necrosis factor-α, insulin-like growth factor-1 (IGF), IGF binding protein-3, 8-isoprostane-F2-alpha, estrone, estradiol, progesterone, sex hormone binding globulin, adiponectin, and leptin.

**Discussion:**

While clinical data indicate that excess weight for height is associated with poor prognosis for long term survival, little attention is paid to weight control in the clinical management of breast cancer. This study will provide information that can be used to answer important patient questions about the effects of dietary pattern and magnitude of weight loss on long term survival following breast cancer treatment.

**Clinical Trial Registration:**

CA125243

## Background

Breast cancer is the most common form of cancer in women in the United States [1] and one of the top ten causes of death [2]. Recent estimates show age-adjusted incidence rate is 123.8 per 100,000 women per year [3]. Body fat is now established as being causally related to postmenopausal breast cancer [1,4-8], with overweight or obese women having almost twice the rates of cancer recurrence and up to 1.5 times the risk of death from breast cancer compared to women in the healthy weight range [1,4-7,9-16]. These are alarming findings since the majority of women in the United States are now overweight or obese (i.e. body weight (kg)/height (m^2^) > 24.9) [17]. This situation is compounded by the fact that weight gain is common post-diagnosis [18,19].

Studies have shown that losing body weight is protective against breast cancer [20,21], and that weight loss can be achieved through multiple approaches [22]. However, it is unclear whether different diets modeled on popular weight loss programs which vary markedly in macronutrient composition differentially affect long term survival following breast cancer treatment [23-35]. Cross sectional, case control and cohort data is conflicting on fat and carbohydrate intake and breast cancer risk [36-40], with little data available about dietary effects on breast cancer survival [41,42]. Moreover, it is not known whether improvement in biomarkers is progressive with increasing weight loss. This could result in very different clinical guidance related to weight loss in these women.

A number of candidate mechanisms including chronic inflammation [43-46], cellular oxidation [47-57], and insulin resistance [58] may explain the link between energy balance and long term survival following breast cancer treatment. Biomarkers that relate to these mechanisms can be measured in blood and urine to assess potential effects. Although maintaining a healthy body weight is protective against breast cancer and weight loss is feasible in post-menopausal breast cancer survivors [59,60], there are no published studies investigating how fat loss using different dietary macronutrient compositions (i.e. dietary patterns) influences these metabolic and hormonal processes. The CHOICE study seeks to address these questions in an effort to strengthen the evidence base on modifiable lifestyle factors, specifically weight loss, and their effects on long term survival following treatment for breast cancer. Typically, weight loss plateaus after 6-months and most weight is regained after 1 to 5 years [61-64], so in addition to determining whether dietary pattern matters, it will also become critical to identify ways to promote weight loss maintenance so that any protection gained is not transient.

## Methods/Design

### Study Design

**Figure 1:**
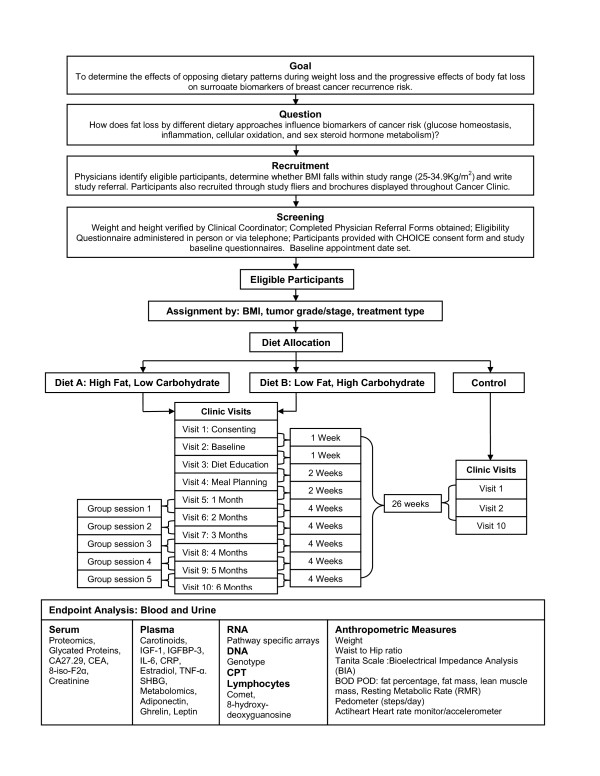
Project Overview

The study, called CHOICE, is a non-randomized, controlled trial in post-menopausal breast cancer survivors investigating whether an energy restricted dietary pattern, i.e. low carbohydrate, high fat or low fat, high carbohydrate, with progressive fat loss during a weight loss program can alter the likelihood of long term survival following treatment for breast cancer as reflected in metabolic and hormonal prognostic biomarkers. A total of 370 women will be accrued and assigned to one of the two dietary intervention arms based on eating preferences or a non-intervention control arm (n = 135 to each intervention arm, n = 100 to the control arm) and followed for 6 months as illustrated in Figure 1. Anthropometric measurements are conducted and biomarkers collected at baseline and 6 months in the control arm, and monthly in the intervention arms in order to model the shape of the metabolic and hormonal response curves.

### Study Aims

#### Primary Aim

The primary aim of the study is to investigate whether dietary pattern (low carbohydrate or low fat) alters the patterns of change observed in circulating factors involved in chronic inflammation, glucose homeostasis, cellular oxidation, and steroid hormone metabolism during progressive loss of body fat. The primary outcome measure is high sensitivity C-reactive protein. All other measurements are considered secondary endpoints.

#### Secondary Aims

1) To explore whether circulating factors associated with glucose homeostasis, chronic inflammation, and cellular oxidation display similar patterns of change in response to progressive fat loss as circulating hormones associated with sex steroid metabolism.

2) To determine whether either dietary pattern has a differential effect on the magnitude or rate of fat loss and lean tissue changes using a number of devices that permit ongoing and accurate measurements of body composition.

### Patient Recruitment (Study Setting and Participants)

CHOICE recruits women who have been treated for breast cancer and attend Rocky Mountain Cancer Centers in Denver, CO, USA. There are approximately 2000 women enrolled in the clinic. Clinic physicians refer breast cancer survivors who meet the eligibility criteria. Participants are also recruited by way of study flyers and brochures posted throughout the clinical facility. Written informed consent is given by subjects when they choose to participate in the program. Following informed consent, participants complete questionnaires that provide demographic, lifestyle and clinical data including age, marital status, ethnicity, level of education, household income, medical history, medicine and supplement use, PAR-Q (Physical Activity Readiness Questionnaire), and emotional well-being. Participants are also interviewed regarding their dieting, weight history and readiness for engaging in a comprehensive weight loss program. This population has a mean age of 60; they are > 98% white and over 50% have post baccalaureate training. While not representative of the general population, this population has proven a very high compliance rate in previous diet-cancer studies conducted by our laboratory [57,65,66].

### Eligibility

Participants must be referred by their clinical oncologist, have a pathology report confirming the resected stage of breast cancer and documentation of the type of systemic adjuvant therapy, as well as have a BMI in the overweight or obese class I range (BMI 25-34.9 kg/m^2^). Eligible participants: must be at least 4 months post radiation or chemotherapy treatment for breast cancer with no evidence of metastatic disease; must not anticipate having surgery over the study duration period; must not follow a special diet excluding foods or food groups; have not lost 2 or more kg of body weight over the month preceding study initiation; must not be taking pharmaceuticals or supplements for weight management; are not being treated for diabetes or blood glucose control; have no history of eating disorders; do not have digestive issues that may interfere with dietary intake, such as irritable bowel syndrome, Crohn's disease, or diverticulitis; have never had surgery involving constriction or removal of any portion of the gastrointestinal tract; have not been diagnosed with hepatitis B, C, or HIV; do not have implanted electronic devices such as a pacemaker; and do not use tobacco products. Participants must also be willing to follow a dietary plan prescribed for the duration of the study; adhere to American Cancer Society alcohol guidelines (≤ 1 standard drink per day); maintain or increase physical activity as prescribed to achieve negative energy balance required for 0.5-1.0 kg weight loss per week; wear a pedometer and record daily activity; wear an accelerometer/heart rate monitor for 2 weeks during the study (1 week at the beginning and 1 week at the end of the study); wear a body or swim suit and cap for body composition testing; record food intake daily; and attend up to ten one-on-one clinic visits and 5 group visits with seven fasting blood and first-void urine samples in the intervention groups, or three one-on-one clinic visits and two fasting blood and first-void urine samples in the control group over 26 weeks.

### Study Groups

#### Control

Individuals accrued to the non-intervention control group are given the same information currently provided to all breast cancer patients about the importance of avoiding post treatment weight gain, and the health benefits of having a BMI in the normal range. Clinical specimens, questionnaire data, body composition, energy expenditure from activity and anthropometric data are collected at the baseline and 6-month (end of study) visits.

### Intervention

Intervention participants follow a fully defined diet-physical activity program designed to create a weekly negative energy balance equivalent to 3500 kcal, after adjustments for metabolic adaptations that occur during extended periods of weight loss. The intervention groups (135 participants in each arm) receive the same physical activity protocol promoting published physical activity guidelines and translated into step recommendations [67,68], but one of two dietary patterns: low fat or low carbohydrate.

### Dietary Plans

Dietary patterns are composed of opposing fat and carbohydrate contents but balanced in protein (Table 1). Six-weeks of meal plans were designed for five calorie levels in each diet arm. The meal plans developed included interchangeable meal options with home-prepared recipes and meal instructions. Supporting materials are provided to facilitate adherence including eating out and frozen meal options, food brand options consistent with the plan, meal planning tools and shopping lists. Educational materials were developed based on a systematic review of strategies supporting weight loss maintenance, incorporating program components (e.g. self-monitoring tools) and core competencies reinforcing weight loss behaviors. These strategies are taught to participants through the one-on-one and group sessions in order to promote high levels of dietary adherence.

**Table 1:** Mean Proposed Macronutrient Composition by Diet Group

	Low Carbohydrate, High Fat	High Carbohydrate, Low Fat
Carbohydrate (%)	32	64
Fat (%)	48	16
Protein (%)	20	20

Participants are instructed to increase their physical activity by increasing steps or step equivalents to contribute to a 500 calorie deficit each day in combination with caloric restriction. Calorie goals are determined based on resting metabolic rate and energy expenditure from activity.

### Outcome Measures

#### C-Reactive Protein

Overweight, obesity and insulin resistance are associated with increases in various cells such as adipocytes, pre-adipocytes, fibroblasts, and macrophages, which release adipokines and promote inflammation [69-71]. The amount of body fat predicts inflammatory C-reactive protein (CRP) levels among adults [72]. Inflammation decreases apoptosis, increases breast cancer invasiveness and decreases prognosis [46,73]. There is convincing evidence that persistent low grade inflammation as well as chronic inflammatory diseases are associated with several cancers in humans [44]. Importantly, inflammation measured via CRP is inversely associated with breast cancer survival, even independently of BMI [36,43]. Therefore, CRP is considered the primary outcome.

### Secondary Outcomes

#### Other Markers of Inflammation

Other inflammatory cytokines that mediate the inflammatory response will also be measured, including Interleukin-6 (IL-6) and TNF-α. IL-6 has been correlated with extent of tumor invasion and metastasis [74].

### Glucose Homeostasis

As hyperglycemia and insulin resistance are associated with an increased risk for breast cancer [75], factors related to these metabolic processes will be measured including homeostasis model assessment (HOMA). HOMA is a method used to quantify insulin resistance and pancreatic beta cell function and is a calculated index based on fasting levels of insulin and glucose, giving an integrated view of glucose utilization. HOMA has been shown to be associated with increased breast cancer incidence [75], and higher breast cancer mortality in the HEAL study [76]. Fasting insulin alone has also been shown to be predictive of survival in breast cancer patients [77]. In addition, insulin-like growth factor-1 (IGF-1), IGF binding protein-3 (IGFBP-3) and the ratio of IGF-1:IGFBP-3, which provides an estimate of biologically available IGF-1, a more useful indication of overall, longer term control of glucose homeostasis in relation to breast cancer risk, will be measured [78]. Serum IGF-1 is positively associated with breast cancer risk, as well as changes in response to caloric restriction and nutritional alterations [79]. Measuring the effects of two differing dietary patterns on endogenous production of IGF-1 will help to further characterize dietary effects on breast cancer risk.

### Cellular Oxidation

The inflammatory response stimulated by obesity and insulin resistance also increases oxidative stress in the body [80]. During oxidative stress, byproducts of nucleic acid metabolism including reactive oxygen species (ROS) promote cancer development by causing genetic mutations and DNA damage [55,56,81]. Secondary measures of cellular oxidation that will be measured include 8-hydroxy-deoxy-guanosine (8-OH-dG) reflecting defects in DNA repair capacity, and markers of whole body lipid peroxidation, 8-isoprostane-F2-alpha (8-iso-PGF_2α_), which have been shown to play a role in breast carcinogenesis [49,82,83]. These byproducts of oxidation are more common in cancerous breast tissue compared to normal breast tissue [49,55,56], and may also be altered with reducing energy intake [51,52] and changes in dietary macronutrient composition [84].

### Hormone Metabolism

Overweight postmenopausal women have elevated concentrations of circulating estrogens and lower concentrations of sex hormone binding globulin (SHBG), which promotes cell growth, putting them at more than twice the risk for breast and endometrial cancers, as evidenced in the Healthy Eating and Lifestyles (HEAL) study and the European Prospective Investigation into Cancer and Nutrition (EPIC) study [12,85,86]. Adipose tissue exhibits aromatase enzyme activity, converting androgenic precursors to estradiol and estrone. Estrogens may promote tumorigenesis through direct or indirect induction of free radical-mediated DNA damage, genetic instability, cell mutations and cell proliferation. Agents such as tamoxifen (selective estrogen receptor modulators) or the aromatase inhibitors have been shown to reduce breast cancer incidence and recurrence [87]. Although warranted, there are currently no published randomized, controlled studies of the effect dietary pattern has on estrogens or SHBG and other candidate mechanisms [88-90]. Secondary hormone metabolism outcomes will include estradiol, estrone, progesterone, and SHBG.

### Adipokines

Adipose tissue produces the hormones adiponectin and leptin. Low adiponectin, which is an anti-inflammatory and insulin sensitizer, is associated with increased breast cancer mortality in breast cancer survivors [76], with leptin regulating energy balance and metabolism and playing a role in cell proliferation [91]. These adipokines, as well as plasma ghrelin, will be measured to provide biological determinants that may also help explain differences in response to the opposing dietary patterns.

### Anthropometry

Weight, height and waist and hip circumferences are measured using a standardized protocol. Participants are in their bathing or body suit for all measurements. Anthropometry is measured monthly in the intervention group and at baseline and 6 months in the control group. Height is measured with a stadiometer. Body circumferences (waist and hip circumferences) are measured using a specially manufactured, tension controlled cloth tape. The participant is instructed to stand erect with arms relaxed at their sides, feet together and abdomen relaxed. Waist circumference is defined as the diameter around the abdomen; it is measured at the midpoint between the top of the hip bone and the bottom of the rib cage. The measurement is taken at the end of a normal expiration, with the tape pulled tight but not compressing the skin. Hip circumference is measured at the level of the maximum extension of the buttocks.

### Body Fat

BOD POD technology is fundamentally the same as underwater (hydrostatic) weighing, but uses volumetric air displacement versus water displacement. All outcome measures including weight are assessed using validated and standardized measuring equipment and techniques. Air displacement plethysmography (BOD POD, Life Measurement, Inc., Concord, CA) has been shown to measure changes in body composition in response to weight change to the same extent as dual x-ray absorptiometry (DEXA), with similar sensitivity [92]. The BOD POD measures the volume of air a person's body displaced while sitting inside a comfortable chamber. By using air versus water, the BOD POD offers a fast, safe, and easy-to-use tool for measuring body composition, without sacrificing accuracy. Since it is based on the same whole-body measurement principle as hydrostatic weighing, the BOD POD first measures the subject's mass and volume. From these measurements, whole-body density is determined, and body fat and lean mass calculated using standardized equations.

### Process Measures

#### Physical Activity

Energy expenditure is measured using accelerometry and heart rate monitoring technology (Actiheart, CamNtech, Inc., Boerne, TX). The Actiheart has been assessed as being valid and reliable in predicting energy expenditure with walking and running [93,94]. Individuals are given a plan to increase physical activity to ultimately meet national guidelines (10,000 steps/d) and facilitate energy balance tailored to their individual lifestyle and physical ability.

### Dietary Adherence

There is often a large loss to follow up in lifestyle interventions, especially in longer term studies, which could cause selection bias due to attrition and over-estimation of treatment effects [95]. One method to optimize adherence and retention is to carefully select participants who are more likely to complete the study. The requirements of completing and returning the screening questionnaires, attendance at the individual meetings, the necessity of a clinic visit and the successful completion of the one week food records and other questionnaires are burdensome on potential participants and result in a lower drop-out rate after assignment to a study arm. Additionally, study coordinators stress to potential participants at every screening visit the various obligations required of them in order to take part in this very structured diet study. Potential participants with doubts about participating will be encouraged not to participate in the study or encouraged to participate in the control arm until they are more prepared to make the necessary commitments to the study. The major method for monitoring adherence to the diet is by the use of daily food record with physical activity/step records. Adherence is promoted by 1) ongoing individual contacts in person and by telephone, mail, and email; 2) continuous care/problem solving treatments; 3) skills training to prevent or cope with setbacks; 4) social support and social influence strategies and 5) ease of recording dietary intake by use of meal codes. Additionally, a program of weight management competencies with ongoing assessment and reinforcement has been developed to target the gaps in participants' knowledge and behaviors related to weight loss. During the screening process, participants will be assessed for their readiness to change or maintain change, as well as their general wellbeing or level of depression, which has been shown to be reciprocally associated with obesity and predict weight maintenance success [96].

### Monitoring Breast Cancer Recurrence

Women with a history of invasive breast cancer are seen every 3 months for the first 2 years following treatment at the study site clinic. At each visit, a clinical history is updated, a physical examination is performed by the attending oncologist, and serum levels of CA 27.29 and CEA are determined. The frequency of clinical visits decreases to every 6 months for the next three years, and follow up occurs annually thereafter. Following a patient's enrollment into this study, and for the duration over which the project is funded, at each of these clinical visits, the relevant disease recurrence data will be recorded along with height, weight, and body composition. Blood will also be drawn and banked at each visit for subsequent hypothesis testing, although such analyses are beyond the scope of this project.

### Data Analysis

All data elements collected will be examined using descriptive statistics (mean, median, SD, range, percentiles, proportion) and graphical summaries (box plots, profile plots by time and diet group). Log transformations will be made before further analysis to stabilize variances as needed.

The primary hypothesis on C-reactive protein will be tested using the following ANCOVA model:

Where Y_4 _is the outcome measure at the end of 6 months, Y_1 _is the outcome measure at baseline, and, GLYC is a 2-level indicator for dietary pattern. This method of analysis adjusts for any remaining pre-treatment differences between groups (a precaution against imbalance after diet assignment) and reduces variability in the data being analyzed [97], thus improving the power of the test for the main effect of interest, b_2_.

There will be a wealth of information in the repeated measures on each subject; the results for all measures using all available data from all time points will be estimated in a mixed-effects repeated measures model to assess the slopes and between group differences after each month of weight loss [98]. The power of this approach lies in its ability to incorporate all of the available longitudinal data even in the unbalanced case, that is, when some of the observations are missing for one or more individuals. Observations within a person over time are allowed to be correlated while observations across individuals are assumed to be independent. These models will also be used to explore the effects of breast cancer stage, BMI (a time-varying covariate) and age.

Depending on the appearance of change over time (seen in the profile plots of each outcome measure by time and diet) linear or nonlinear mixed models will be used. If the trend appears to be linear, the following model for the response vector ***y***_i _for the *i*th group will be used:(1)

where **b**_*i *_~ *N *(0,D) and **e**_*i *_~ *N *(0,R_*i*_) are independent. X_*i *_is a fixed effects design matrix that includes indicators for diet group (1, 2), assessment time (after each 1.5 kg fat loss, and potential covariates (age) or confounders (disease stage, BMI). **Z**_*i *_is a design matrix for the random effects that allows random subject deviations from the population average response. The marginal distribution of **y**_*i *_is normal with mean **X**_*i *_*β *and variance . Parameter estimation in SAS allows a wide range of specifications for the forms of **D **and **R**, and combines empirical Bayes and restricted maximum likelihood using the EM algorithm.

If the descriptive graphs suggest a nonlinear model is appropriate, we will estimate:(2)

Where **b**_*i *_~ *N *(**0**,**D**) and **e**_*i *_~ *N *(**0**,**R**_*i*_). The marginal distribution of **y**_*i *_is difficult to find in most cases, but its mean and variance can be approximated by

Where  is the partial derivative of *f *(⋅) with respect to **b**_*i*_. Parameter estimation in SAS combines a linearization algorithm, such as Gauss-Newton, and the method of Laird-Ware for linear mixed models. We will explore the use of nonlinear models only if it appears that the response trajectory of *Y *over time could be fit well by a smooth nonlinear function. Otherwise, simpler piece-wise linear mixed models will be fit using Equation 1 above.

Secondary measures in the 4 families of outcomes will be assessed using the same statistical methods described above for the primary measures. That is, ANCOVA assessment of the measure by diet group at 6 months followed by an exploratory analysis with mixed models using all available data.

Mixed models will also be used to estimate the effects of fat loss on the 4 families of outcomes. Fat loss and weight loss will be modeled with the expectation that differences across diets will be minimal, and success in weight loss better explained by age, initial BMI, and usual level of physical activity.

Missing data are expected to be missing at random, and are unlikely to exceed 5% of all observations. This assumption will be checked during the initial descriptive analysis of the data after database lock, and appropriate sensitivity analyses will be done if there is evidence that the data are MNAR (missing not-at-random) [99]. Given the nature of the patient population and the incentives to return for monthly evaluations, we expect to collect most endpoint measures regardless of compliance. We will accrue 370 subjects with the expectation of completing at least 135 per diet group and 100 in the control group, that is, 10% loss to follow-up. Analysis will be intent-to-treat. All statistical analysis will be done using SAS version 9 (SAS Institute, Cary, NC).

### Limitations

Limitations include the fact that the study is neither double blinded nor randomized; results may not be generalizable. The population is free living and as such is subject to issues of compliance and difficulties with quantifying dietary intake and physical activity.

## Discussion

A number of reports indicate that the prognosis for long term survival following treatment for breast cancer is better in women whose body weight for height, assessed by body mass index (BMI, body weight (kg)/height (m^2^)), is considered to be in the normal range (BMI 18.5 to 24.9) versus women who are overweight (BMI 25.0 to 29.9) or obese BMI ≥ 30.0 [1,4,6,7,9-13,100,101]. Consistent with those reports is the observation that weight gain post diagnosis increases risk for breast cancer recurrence; whereas, weight loss in breast cancer survivors improves the chances of long term survival [20,21]. If one takes the available epidemiological and clinical data at face value, it prompts the question of why relatively little attention is paid to weight control in the clinical management of breast cancer survivors post treatment.

Overweight and obesity are common problems in the United States, and there is little evidence to indicate that prevalence is less in breast cancer survivors than in the population at large, which is estimated to be > 60% [17]. Obesity has been reported to be the cause for at least 9% breast cancer cases [102]. Thus, given that the majority of breast cancer survivors have excess weight as a risk factor, the population at risk is large. However, a number of challenges are faced by the physician. They include issues such as initiating a conversation about weight loss while recognizing the sensitivity of the subject and time constraints of office visits, which do not allow sufficient time to address the complexity of individual weight management issues, including the knowledge and behavioral gaps related to diet and weight loss. Moreover, there may be hesitation to emphasize weight loss given the recognized 95% long term failure rates of most weight control efforts, making this information a lower priority during the office visit [22,103-110]. Additionally, due to lack of knowledge about the subject matter, basic questions such as, 'How should weight loss be achieved?' and, 'How much weight loss will provide benefit?' cannot be answered with confidence. While many studies have examined differences in effectiveness among various approaches to weight loss [22,103-110], relatively few studies have been conducted in a free living population of breast cancer survivors in the private practice setting. This study will provide information that can be used to answer patient's questions about the effects of dietary pattern and magnitude of weight loss on long term survival following breast cancer treatment.

## List of abbreviations

BMI: body mass index; CA 125: cancer antigen 125; CEA: carcinoembryonic antigen; CRP: C-reactive protein; DEXA: dual-energy x-ray absorptiometry; EPIC: European Prospective Investigation into Cancer and Nutrition; HEAL: Health, Eating, Activity and Lifestyle study; HOMA: homeostatic model assessment; IGF: insulin-like growth factor; IGF-BP: insulin-like growth factor binding protein; IL: interleukin; MNAR: missing not-at-random; PAR-Q: physical activity readiness questionnaire; ROS: reactive oxygen species; SHBG: sex hormone binding globulin; TNF-α: tumor necrosis factor alpha; 8-OHdG: 8-hydroxydeoxyguanosine; 8-ISOPGF2α: 8-isoprostaglandin F2 alpha.

## Competing interests

The authors declare that they have no competing interests.

## Authors' contributions

HJT, SMS, PW, JNM, and MRW participated in the design and implementation of the study. MCP, EAD, WJ, ZZ participated in the implementation of the study. All authors participated in the preparation of the manuscript. All authors have read and approved the final manuscript.

## Pre-publication history

The pre-publication history for this paper can be accessed here:

http://www.biomedcentral.com/1471-2407/11/287/prepub
